# Skin Autofluorescence Is Associated with Diabetic Peripheral Neuropathy in Chinese Patients with Type 2 Diabetes: A Cross-Sectional Study

**DOI:** 10.1089/gtmb.2018.0328

**Published:** 2019-05-30

**Authors:** Li Wan, Guijun Qin, Wenhao Yan, Tongwen Sun

**Affiliations:** ^1^ Department of Comprehensive ICU, First Affiliated Hospital of Zhengzhou University, Zhengzhou, China.; ^2^ Endocrine Department, First Affiliated Hospital of Zhengzhou University, Zhengzhou, China.; ^3^ Pediatric Medicine, First Affiliated Hospital of Zhengzhou University, Zhengzhou, China.

**Keywords:** diabetic peripheral neuropathy, skin autofluorescence, nerve conduction studies, type 2 diabetes

## Abstract

***Background:*** Diabetic peripheral neuropathy (DPN) affects nearly 50% of the diabetic population. Advanced glycation end products, measured through skin autofluorescence (SAF), play an important role in the diagnosis and prevention of DPN. To date, however, no relevant study has discussed the relationship between SAF and the Chinese population.

***Objective:*** We conducted this study to evaluate the association between DPN and SAF among the Chinese population.

***Methods:*** In this cross-sectional study, we recruited a total of 820 patients with type 2 diabetes. All of the patients underwent SAF measurements and a nerve conduction study (NCS). Post-SAF characterization, the patients were divided into three groups according to the first and third quartiles of their SAF values (AU) (SAF ≤ 2.2; 2.2 < SAF ≤ 2.7; SAF > 2.7). Based on the results of the NCS, patients were divided into two groups: DPN and non-DPN.

***Results:*** When compared with the non-DPN group (*n* = 275) with the DNP group. The latter had higher SAF values (2.72 ± 0.55 AU vs. 2.17 ± 0.71 AU, *P* < 0.01). There were significant differences in age, the percentage of DPN, and NCS parameters, including motor nerve conduction velocity, sensory nerve conduction velocity, distal latency, and sensory nerve action potential among the three SAF groups (*p* < 0.05). The SAF value was positively associated with DPN (*r* = 0.11, *p* < 0.01). After adjusting for all potential confounders, the SAF values were still associated with an increased risk of DPN (odds ratio 5.15; 95% confidence interval [1.48–4.53]) (*p* < 0.01). A receiver operating characteristic analysis indicated that an SAF value >2.57 ng/mL predicts a threefold increased risk of DPN (*p* < 0.01).

***Conclusions:*** SAF is an independent risk factor for DPN, which might be of potential value for screening DPN in Chinese patients with type 2 diabetes.

## Introduction

Diabetic peripheral neuropathy (DPN) is one of the most common complications arising from diabetes, affecting approximately half of the patients during the course of the disease. In addition, DPN is an independent risk factor for foot ulcers and lower extremity amputations, which leads to a decline in quality of life and poses huge challenges to society (Argoff *et al.*, 2006; Icks *et al.*, 2009). The precise mechanism leading to DPN remains unclear; however, it does involve hyperglycemia, protease C activation, and oxidative stress (Tesfaye *et al.*, 2005; Callaghan *et al.*, 2012; Naziroglu *et al.*, 2012; Menne *et al.*, 2013).

In recent years, the aggregation of advanced glycation end products (AGEs) has attracted increased attention in studies related to the pathogenesis of DPN (Meerwaldt *et al.*, 2005; Toth *et al.*, 2008; Park *et al.*, 2014). AGEs are the end products of nonenzymatic reactions between sugars and proteins. The aggregation of AGEs is reported to be related to chronic hyperglycemia and oxidative stress. It was also revealed, through skin biopsies in a diabetes control and complications trial, that the AGEs level in the skin tissue of type 1 diabetes patients with DPN was higher than in non-DPN patients (Monnier *et al.*, 1999). Inhibiting the aggregation of AGEs can slow down the development of diabetic complications, including DPN and diabetic macroangiopathy (Cameron *et al.*, 1992).

It is well proven that an autofluorescence reader (AFR) can noninvasively detect skin autofluorescence (SAF) and reflects the degree of accumulation of AGEs in skin tissue. Previous studies have investigated the relationship between SAF and diabetic complications. In one study, when diabetic patients were compared with healthy controls, they had significantly increased levels of SAF (Meerwaldt *et al.*, 2004). SAF has been reported to play a role in screening for diabetic microvascular complications and early stage atherosclerosis (Yoshioka, 2018). It has been shown that SAF is significantly correlated with DPN and plays a role in the diagnosis and prevention of DPN (Gerrits *et al.*, 2008; Rajaobelina *et al.*, 2017). However, the diagnosis of DPN has often lacked objectivity and consistency.

Nerve conduction studies (NCSs) are currently regarded as the gold standard for a diagnosis of DPN and are widely used in clinical evaluations. There are no relevant studies that have examined this relationship between SAF and DPN in the Chinese population. We conducted this study to clarify the connection between SAF and DPN, as well as to assess the value of this noninvasive measurement for DPN screening in Chinese patients with type 2 diabetes.

## Patients and Methods

### Study population

From December 2016 to November 2017, we recruited 820 patients with type 2 diabetes at the First Affiliated Hospital of Zhengzhou University for this cross-sectional study. The inclusion criteria included the 1999 World Health Organization criteria and the 2012 American Diabetes Association standards (American Diabetes, 2012). The exclusion criteria included type 1 or specific types of diabetes mellitus, acute complications of diabetes, renal dysfunction (glomerular filtration rate [GFR] <60 mL/min per 1.73 m^2^), osteomalacia, a history of cerebral infarction, and degenerative changes in the cervical vertebrae. This study was approved by the Human Research and Ethics Committee of the First Affiliated Hospital of Zhengzhou University and adhered to the tenets of the Declaration of Helsinki. All of the patients signed the informed consent.

### Physical examination

A questionnaire was used to obtain the following information: gender, age, duration of diabetes, and history of smoking and drinking. Height, weight, blood pressure (averaged three times' values), and waist and hip circumference were measured using a standardized method by the same nurse. Body mass index = weight/height^2^ (kg/m^2^); waist:hip ratio = waist circumference/hip circumference.

### Laboratory measurements

Blood samples were collected from an antecubital vein. Two-hour postmeal blood glucose (2hPG) and fasting plasma glucose were performed by the glucose oxidase method (Wako pure 2756-01; Wako Pure Chemical Industries, Osaka, Japan). An automatic biochemical analyzer using an enzymatic method was used to determine total cholesterol (TC), triglyceride (TG), high-density lipoprotein cholesterol (HDL-C), low-density lipoprotein cholesterol (LDL-C), blood urea nitrogen (BUN), serum creatinine (Cr), and uric acid (UA) levels. Glycosylated hemoglobin (HbA1c) and glycosylated serum albumin (GA) were estimated by high-pressure liquid chromatography (Variant™II machine; Bio-Rad, Hercules, CA) and a liquid enzymatic method (MD, Inc., Silicon Valley, CA), respectively. Urinary albumin was determined by RIA and GFR was evaluated by technetium-99m diethyl triamine penta-acetic acid clearance.

### SAF detection

SAF was determined by an AFR (DiagnOptics BV, Groningen, the Netherlands). Using the AFR with an excitation light source of 300–420 nm, fluorescence of the skin was measured on the arm. Autofluorescence was defined as the average fluorescence per nanometer over the entire emission spectrum (420–600 nm) as a ratio of the average fluorescence per nanometer over the 300–420 nm range (Meerwaldt *et al.*, 2004). Post-SAF detection, the patients were divided into three groups according to the first and third quartile of the SAF value (AU) (SAF ≤ 2.2; 2.2 < SAF ≤ 2.7; SAF > 2.7).

### Neuropathy assessment

A NCS was conducted by electromyogram (Myto, EBNeuro, Firenze, Italy) in a quiet room by the same technician. The motor nerve assessment included the measurement of motor nerve conduction velocities (MNCVs), compound muscle action potential, amplitude and distal latency (DL) of the median, ulnar, tibial, and common peroneal nerve. The sensory nerve assessment included measurements of sensory nerve conduction velocities (SNCVs) and sensory nerve action potentials (SNAP) of the median, ulnar nerve, sural, and superficial peroneal nerve. An infrared lamp was used to maintain a skin temperature >31°C during all the tests.

Abnormal nerve conduction function was defined as an abnormality of more than one examination parameter of two or more nerves (including at least one lower limb nerve) (Dyck *et al.*, 2011). DPN was defined as diabetic patients with neurotransmitter dysfunction. Based on the results of the neuropathy assessment, patients were divided into two groups: DPN group and non-DPN group.

### Statistical analysis

Data were analyzed and processed using SPSS 22.0 software. For continuous variables, differences were analyzed by Student's or chi-square test as appropriate. For categorical values, a comparison was performed by the chi-squared test. Logistic regression analysis was employed to evaluate the odds ratio (OR) in three logistic regression models: a nonadjusted model; an age and gender-adjusted model; and a multivariable model adjusted for all variables, with *p* < 0.05 in the nonadjusted model. Receiver operating characteristic (ROC) curve was demonstrated to evaluate the screening value of SAF to DPN. Two-tailed *p*-values <0.05 were considered statistically significant.

## Results

### Comparison of clinical features between DPN and non-DPN groups

We recruited 820 patients, including 430 men and 390 women, with a mean age of 60.72 ± 10.23 years and mean duration of 12.77 ± 8.08 years. Significant differences were demonstrated for SAF, age, duration of disease, glucose control, and renal function between the two groups of patients (all *p* < 0.05, Table 1). The mean value of SAF in the DPN group was significantly higher than that in non-DPN group (2.72 ± 0.55 AU vs. 2.17 ± 0.71 AU, *p* < 0.01).

**Table 1.** Characteristics of the Study Populations

	*Without DPN (*n* = 547)*	*With DPN (*n* = 273)*	p
No. of cases (men/women)	277/270	153/120	0.154
Age (years)	58.36 ± 11.91	65.77 ± 9.31	0.032
Diabetes duration (years)	9.51 ± 7.01	14.18 ± 7.55	0.002
SBP (mmHg)	133.29 ± 15.69	135.17 ± 14.14	0.518
DBP (mmHg)	77.26 ± 8.02	78.92 ± 8.98	0.398
Height (cm)	161.92 ± 7.21	163.53 ± 6.25	0.229
Weight (kg)	68.77 ± 10.73	70.11 ± 10.12	0.447
BMI (kg/m^2^)	25.15 ± 2.67	25.29 ± 3.01	0.455
Waistline (cm)	89.43 ± 8.78	90.24 ± 7.89	0.659
Hipline (cm)	98.65 ± 7.21	98.48 ± 6.98	0.983
Waist:hip ratio	0.93 ± 0.07	0.93 ± 0.07	0.898
Smoking (%)	33.31	33.30	0.821
Drinking (%)	20.57	19.87	0.822
FBG (mM)	8.26 ± 2.31	8.60 ± 3.09	0.118
2hPG (mM)	16.63 ± 6.11	17.41 ± 6.03	0.070
HbA1c (%)	7.42 ± 1.39	8.47 ± 1.90	0.004
GA (%)	15.05 ± 2.88	15.74 ± 2.04	0.061
TC (mM)	1.16 ± 0.31	1.39 ± 0.33	0.410
TG (mM)	1.61 ± 0.82	1.83 ± 0.78	0.072
HDL-C (mM)	2.19 ± 2.06	2.01 ± 1.76	0.130
LDL-C (mM)	1.60 ± 1.09	1.42 ± 1.87	0.962
BUN (mM)	6.32 ± 1.77	6.94 ± 1.52	0.551
Cr (μM)	67.10 ± 15.93	68.17 ± 17.46	0.402
UA (μM)	328.38 ± 61.44	321.97 ± 69.08	0.421
GFR (mL/min/1.73 m^2^)	92.99 ± 24.51	84.81 ± 23.32	0.002
Urinary albumin (mg/24 h)	105.05 ± 7.68	226.32 ± 10.29	0.001
SAF (AU)	2.17 ± 0.71	2.72 ± 0.55	0.000

DPN, diabetic peripheral neuropathy; SBP, systolic blood pressure; DBP, diastolic blood pressure; BMI, body mass index; FBG, fasting blood glucose; 2hPG, 2-hour post-meal blood glucose; HbA1c, glycosylated hemoglobin; GA, glycosylated serum protein; TC, total cholesterol; TG, triglyceride; HDL-C, high-density lipoprotein cholesterol; LDL-C, low-density lipoprotein cholesterol; BUN, blood urea nitrogen; Cr, serum creatinine; UA, uric acid; GFR, glomerular filtration rate; SAF, skin autofluorescence.

### Comparison of NCS among three SAF groups

As previously stated, the patients were additionally divided into three groups according to their SAF values (AU) (SAF ≤ 2.2, *n* = 287; 2.2 < SAF ≤ 2.7, *n* = 369; SAF > 2.7, *n* = 164). The differences in the NCS parameters among the three SAF groups are summarized in Table 2. There were significant differences among the three SAF groups (all *p* < 0.05) for age; percentage of individuals with DPN; TC levels; MNCV of the median, peroneal, and tibial nerve; DL of the tibial nerve; SNAP of median, ulnar, and superficial peroneal nerve; and SNCV of the sural nerve and superficial peroneal nerve. The patients with higher SAF value were shown to be accompanied with slower NCS according to Table 2.

**Table 2.** Characteristics of Patients with Different Skin Autofluorescence Level

	*SAF ≤ 2.2*	*2.2 < SAF ≤ 2.7*	*SAF > 2.7*	p
No. of cases (men/women)	151/136	189/180	88/76	
Age (years)	56.76 ± 11.21	58.31 ± 11.91^*^	65.70 ± 10.32^#^	0.014
Diabetes duration (years)	12.22 ± 6.54	14.18 ± 7.17	13.62 ± 7.89	0.152
SBP (mmHg)	132.33 ± 12.17	134.89 ± 14.56	135.81 ± 13.77	0.212
DBP (mmHg)	76.96 ± 9.11	78.43 ± 8.32	79.15 ± 9.01	0.561
HbA1c (%)	7.94 ± 1.39	8.49 ± 1.90	7.22 ± 1.78	0.356
DPN (%)	57 (19.86%)	121 (32.79%)^**^	95 (57.92%)^##^	0.002
TC (mM)	1.21 ± 0.49	1.32 ± 0.55^**^	1.41 ± 0.61^##^	0.010
TG (mM)	2.59 ± 0.77	2.67 ± 0.82	2.85 ± 0.67	0.061
HDL-C (mM)	2.18 ± 1.66	2.11 ± 0.99	2.09 ± 1.21	0.241
LDL-C (mM)	1.46 ± 1.11	1.52 ± 1.02	1.61 ± 1.33	0.672
MNCV median nerve (m/s)	54.65 ± 4.51	53.31 ± 4.24^**^	49.11 ± 5.79^##^	0.000
DL median nerve (ms)	3.67 ± 0.63	3.74 ± 0.72	3.73 ± 0.46	0.973
CMAP median nerve (mV)	10.47 ± 2.88	10.12 ± 2.93	9.77 ± 3.14	0.223
MNCV ulnar nerve (m/s)	57.19 ± 7.62	56.33 ± 6.12	55.09 ± 6.77	0.532
DL ulnar nerve (ms)	2.41 ± 0.77	2.59 ± 0.62	2.89 ± 0.31	0.176
CMAP ulnar nerve (mV)	12.03 ± 3.12	11.98 ± 2.63	12.77 ± 3.01	0.389
MNCV peroneal nerve (m/s)	47.79 ± 4.87	45.28 ± 4.67^**^	41.02 ± 3.62^##^	0.000
DL peroneal nerve (ms)	3.62 ± 0.62	3.66 ± 0.79	4.06 ± 0.81	0.171
CMAP peroneal nerve (mV)	6.11 ± 2.98	6.03 ± 3.33	5.97 ± 2.27	0.229
MNCV tibial nerve (m/s)	45.72 ± 5.32	42.77 ± 4.82^**^	39.02 ± 4.92^##^	0.007
DL tibial nerve (ms)	4.17 ± 0.72	4.53 ± 1.01	5.12 ± 0.91	0.020
CMAP tibial nerve (mV)	13.02 ± 5.16	13.91 ± 5.88	12.67 ± 6.01	0.373
SNCV median nerve (m/s)	52.14 ± 8.30	51.74 ± 7.91	50.22 ± 8.79	0.372
SNAP median nerve (mV)	26.12 ± 7.21	22.31 ± 6.92^*^	17.79 ± 7.03^#^	0.032
SNCV ulnar nerve (m/s)	57.19 ± 5.77	56.42 ± 5.72	56.97 ± 5.15	0.426
SNAP ulnar nerve (mV)	16.77 ± 7.39	14.12 ± 7.77	12.90 ± 5.12	0.041
SNCV sural nerve (m/s)	51.81 ± 4.26	48.33 ± 4.95^*^	41.33 ± 4.12^#^	0.005
SNAP sural nerve (mV)	13.02 ± 5.44	8.23 ± 4.75^*^	7.01 ± 4.32^#^	0.022
SNCV superficial peroneal nerve (m/s)	52.74 ± 8.21	46.15 ± 6.88^*^	41.05 ± 6.23^#^	0.012
SNAP superficial peroneal nerve (mV)	11.17 ± 4.78	9.29 ± 5.08^*^	6.81 ± 4.57^#^	0.026

^*^ *p* < 0.05, ^**^p < 0.01 versus low SAF group (SAF ≤ 2.2).

# *p* < 0.05, ^##^*p* < 0.01 versus medium SAF group (2.2 < SAF ≤ 2.7).

MNCV, motor nerve conduction velocity; DL, distal latency; CMAP, compound muscle action potential; SNCV, sensory nerve conduction velocity; SNAP, sensory nerve action potential.

### Association of variables with DPN

SAF, age, duration, 2hPG, TC, LDL-C, and BUN levels were negatively associated with DPN (Table 3, all *p* < 0.05). Table 4 indicates the factors associated with DPN in three statistical models. First, in the nonadjusted model, there were significant associations between DPN, and SAF, age, duration, 2hPG, HbA1c, TC, and LDL-C (all *p* < 0.05). After adjusting for age and gender, the association between DPN, and SAF (OR 5.62, 95% confidence interval [CI] 2.23–8.96), duration (OR 1.03, 95% CI 0.73–1.33), 2hPG (OR 2.67, 95% CI 1.02–3.75), and LDL-C (OR 1.07, 95% CI 1.04–1.09) still existed (all *p* < 0.05). In the multivariable model, SAF (OR 5.15, 95% CI 1.88–8.67; *p* = 0.02) was still significantly associated with DPN. Spearman's correlation analysis of variables with SAF showed that age, duration, 2hPG, and TC level were positively associated with SAF (Supplementary Table S1, all *p* < 0.05).

**Table 3.** Spearman's Correlation Analysis of Variables with Diabetic Peripheral Neuropathy

*Variable*	*DPN*	
	r	p
Age	−0.28	0.000^**^
Duration of diabetes	−0.13	0.000^**^
SBP	0.03	0.321
DBP	−0.01	0.792
Height	0.01	0.621
Weight	−0.06	0.533
BMI	0.04	0.627
Waistline	0.01	0.412
Hipline	−0.02	0.219
Waist:hip ratio	0.01	0.792
Smoking	−0.02	0.564
Drinking	−0.02	0.541
FBG	0.24	0.199
2hPG	−0.24	0.017^*^
HbA1c	−0.11	0.132
GA	−0.06	0.079
TC	−0.02	0.013^*^
TG	0.05	0.532
HDL-C	−0.06	0.104
LDL-C	−0.04	0.022^*^
BUN	−0.05	0.029^*^
Cr	−0.06	0.071
UA	0.01	0.092
GFR	0.03	0.376
Urinary albumin	0.09	0.215
SAF	−0.11	0.002^**^

^*^ *p* < 0.05, ^**^p < 0.01.

**Table 4.** Association of Variables with Diabetic Peripheral Neuropathy

*Outcome*	*Nonadjusted model*			*Age- and gender-adjusted model*			Multivariate model^*^		
	*OR*	*95% CI*	p	*OR*	*95% CI*	p	*OR*	*95% CI*	p
Diabetes duration	1.13	1.02–1.24	<0.01	1.12	1.01–1.23	<0.01	1.12	1.02–1.22	0.03
Age	1.12	1.01–1.21	<0.01	—	—	—	—	—	—
2hPG	3.48	2.51–4.49	<0.01	2.67	1.02–3.75	0.04	1.84	1.00–2.64	0.04
HbA1c	2.02	1.04–3.12	0.04	—	—	—	—	—	—
TC	1.98	1.03–2.69	<0.01	—	—	—	—	—	—
LDL-C	1.24	1.44–1.65	<0.01	1.07	1.04–1.09	<0.01	1.06	1.02–1.09	<0.01
SAF	6.22	2.49–9.96	<0.01	5.62	2.23–8.96	0.02	5.15	1.88–8.67	0.02

^*^ Variables with *p* < 0.05 in univariate analysis were included in the multivariate model.

CI, confidence interval; OR, odds ratio.

### The screening value of SAF for DPN

**FIG. 1.**
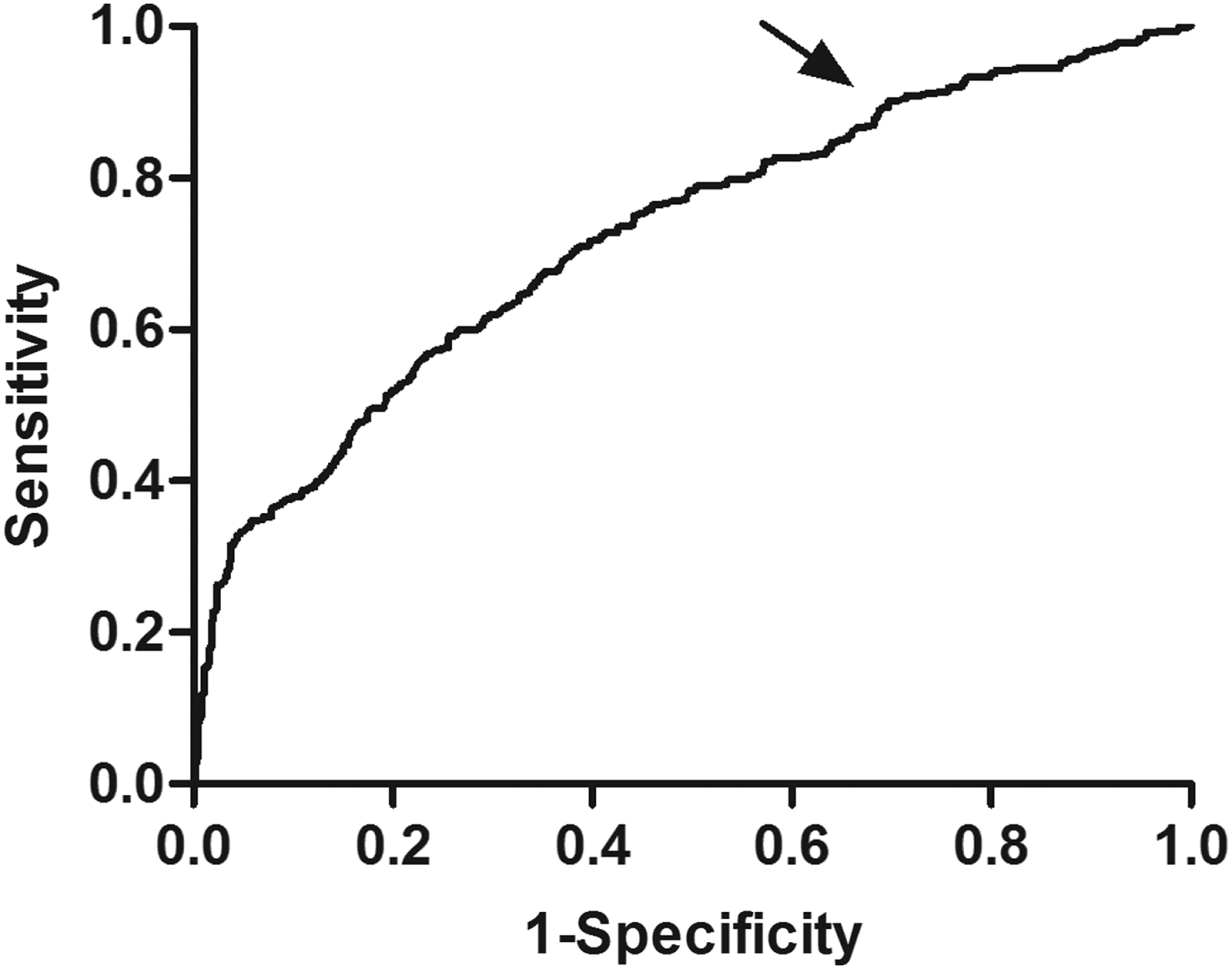
Receiver operating characteristic curve analysis of SAF value for diabetic peripheral neuropathy. AUC = 0.723 (*p* = 0.001), 95% CI: 0.697–0.748. Identified SAF cut point (*arrow*) = 2.57 AU, Youden index = 0.32. Sensitivity: 66.21%; specificity: 65.79%. SAF, skin autofluorescence.

ROC analysis revealed that the optimal SAF cutoff value was 2.57 AU. At this level, the Youden index was 0.32 with a sensitivity of 66.21% and specificity of 65.79% (Fig. 1). The OR value for SAF > 2.57 AU was 3.77 (95% CI 2.51–5.05; *p* < 0.01) for occurrence of DPN.

## Discussion

AGEs aggregation has been regarded as an important pathogenesis factor toward the development of DPN (Meerwaldt *et al.*, 2004; Vincent *et al.*, 2011). In this study, we assessed the relationship between SAF and DPN in a cross-sectional study of the Chinese population with type 2 diabetes. SAF proved to be not only an independent risk factor for DPN but to also have a prognostic value.

The risk factors for DPN identified in our search were consistent with previous studies, including SAF, duration of diabetes, 2hPG, and LDL-C. However, as far as we know, this study is the first to illustrate the screening value of a high SAF level for DPN in Chinese diabetic patients. The close relationship between SAF and DPN has been discussed in other cross-sectional studies in patients with type 1 diabetes in the Japanese population (Araszkiewicz *et al.*, 2016; Yoshioka, 2018). It has been demonstrated that SAF is closely associated with diabetic microangiopathy, including DPN, and could be an important diagnostic and/or predictive indicator of DPN (Monnier *et al.*, 1999; Rajaobelina *et al.*, 2017). In previous studies, however, the diagnostic criteria of DPN were not consistently applied. In addition, the evaluations of DPN were mostly based on 10g single fiber nylon filament tests (Lutgers *et al.*, 2006; Noordzij *et al.*, 2012), vibration threshold test, or even only typical neuropathy symptoms (Rajaobelina *et al.*, 2017). In this study, DPN was diagnosed with NCS results that were objective and reliable. The relationship between the NCS parameters and SAF was further analyzed. It showed that the nerve conduction velocity and the nerve conduction amplitude were significantly lower in the high and medium SAF groups than those in the low SAF group. SAF has also been reported to be negatively correlated with nerve conduction velocity and nerve conduction amplitude in healthy volunteers and diabetic patients without DPN; this indicates that an increase in SAF levels could occur before clinical symptoms of DPN, and the determination of increased SAF could be used for screening for risk of DPN in patients with or without diabetes (Meerwaldt *et al.*, 2005). In addition, our study revealed that motor nerve and sensory nerve conduction function both declined more in the high and medium SAF groups than in the low SAF group. This suggests that the SAF levels are reflective of the degree of nerve function injury in a dose-response manner. In a 4-year longitudinal cohort study, Rajaoblina *et al.* (2017) evaluated peripheral nerve sensitivity, neuropathic pain, autonomic nervous function and muscle strength by vibration perception threshold, DN4 scores, by SUDOSCAN assessment and dynamometer, respectively, showing significant differences between patients with increased SAF levels (SAF > 2.4 AU), and patients with decreased SAF levels (SAF ≤ 1.75 AU). In summary, it appears as if SAF levels accurately reflect the pathological changes of peripheral nerve in diabetic patients, and the results of our ROC analyses show a dose–response relationship between SAF levels and for DPN, which suggests that it could be used as a prognostic indicator.

Some limitations of this study should also be noted. First, DPN was diagnosed by NCS results, which are considered to be a shortage of evaluating small fiber sensory neuropathies. Second, the study population was solely Chinese patients with type 2 diabetes. Finally, the value of SAF in predicting DPN could not be confirmed in this cross-sectional study, which requires further follow-up.

## Conclusions

This cross-sectional study included 820 patients with type 2 diabetes. Post-SAF detection and DPN diagnosis, we found that SAF was an independent factor for DPN and might serve as a screen for the risk of DPN in Chinese diabetic patients.

## Supplementary Material

Supplemental data
