# Supplementary material for: Skin Autofluorescence Is Associated with Diabetic Peripheral Neuropathy in Chinese Patients with Type 2 Diabetes: A Cross-Sectional Study
Source: Genet Test Mol Biomarkers. 2019 May 30;23(6):387–92. doi: 10.1089/gtmb.2018.0328 (PMC6555182; doi:10.1089/gtmb.2018.0328)
Supplement: Supplemental data [file Supp_TableS1.pdf]

Supplementary Data

Supplementary Table S1. Spearman's Correlation Analysis of Variables with Skin Autofluorescence

	SAF	
Variable	r	p
Age	0.62	0.000**
Duration of diabetes	0.27	0.000**
SBP	–0.02	0.715
DBP	0.01	0.218
DPN	–0.11	0.002
Height	–0.01	0.553
Weight	0.07	0.912
BMI	–0.04	0.334
Waistline	–0.01	0.341
Hipline	0.07	0.427
Waist:hip ratio	–0.02	0.452
Smoking	0.12	0.764
Drinking	0.08	0.221
FBG	–0.21	0.345
2Hpg	0.43	0.023*
HbA1c	0.17	0.519
GA	0.05	0.128
TC	0.12	0.000*
TG	0.06	0.197
HDL-C	0.02	0.891
LDL-C	0.02	0.067
BUN	0.11	0.609
Cr	0.11	0.289
UA	–0.01	0.132
GFR	–0.16	0.765
Urinary albumin (mg/24 h)	–0.11	0.341

*$p < 0.05$, **$p < 0.01$.

SAF, skin autofluorescence; SBP, systolic blood pressure; DBP, diastolic blood pressure; BMI, body mass index; FBG, fasting blood glucose; 2hPG, 2-hour post-meal blood glucose; HbA1c, glycosylated hemoglobin; GA, glycosylated serum protein; TC, total cholesterol; TG, triglyceride; HDL-C, high-density lipoprotein cholesterol; LDL-C, low-density lipoprotein cholesterol; BUN, blood urea nitrogen; Cr, serum creatinine; UA, uric acid; GFR, glomerular filtration rate.
